# Gollop–Wolfgang Complex Is Associated with a Monoallelic Variation in *WNT11*

**DOI:** 10.3390/genes15010129

**Published:** 2024-01-20

**Authors:** Adrian Odrzywolski, Beyhan Tüysüz, Philippe Debeer, Erika Souche, Arnout Voet, Boyan Dimitrov, Paulina Krzesińska, Joris Robert Vermeesch, Przemko Tylzanowski

**Affiliations:** 1 Laboratory for Cytogenetics and Genome Research, Department of Human Genetics, KU Leuven, B-3000 Leuven, Belgium; 2 Department of Biochemistry and Molecular Biology, Medical University of Lublin, 20-093 Lublin, Poland; 3 Department of Pediatric Genetics, Cerrahpasa Faculty of Medicine, Istanbul University-Cerrahpasa, 34098 Istanbul, Turkey; 4 Locomotor and Neurological Disorders, Department of Development and Regeneration, KU Leuven, B-3000 Leuven, Belgium; 5 Laboratory of Biomolecular Modelling and Design, Department of Chemistry, KU Leuven, 3001 Heverlee, Belgium; 6 Clinical Sciences, Research Group Reproduction and Genetics, Centre for Medical Genetics, Centre for Medical Genetics, Universitair Ziekenhuis Brussel (UZ Brussel), Vrije Universiteit Brussel (VUB), 1090 Brussels, Belgium; 7 Laboratory of Molecular Genetics, Medical University of Lublin, 20-093 Lublin, Poland; 8 Skeletal Biology and Engineering Research Center, Department of Development and Regeneration, KU Leuven, B-3000 Leuven, Belgium

**Keywords:** Gollop–Wolfgang complex, whole genome sequencing, split-hand/foot malformation, *WNT11*

## Abstract

Gollop–Wolfgang complex (GWC) is a rare congenital limb anomaly characterized by tibial aplasia with femur bifurcation, ipsilateral bifurcation of the thigh bone, and split hand and monodactyly of the feet, resulting in severe and complex limb deformities. The genetic basis of GWC, however, has remained elusive. We studied a three-generation family with four GWC-affected family members. An analysis of whole-genome sequencing results using a custom pipeline identified the *WNT11* c.1015G>A missense variant associated with the phenotype. In silico modelling and an in vitro reporter assay further supported the link between the variant and GWC. This finding further contributes to mapping the genetic heterogeneity underlying split hand/foot malformations in general and in GWC specifically.

## 1. Introduction

Split hand/foot malformation (SHFM) or ectrodactyly is a rare disorder affecting the central rays of the hands and/or feet [1]. The clinical features of SHFM are highly variable and, in most cases, asymmetrical (MIM #183600, #220600, #225300, #246560, #313350, #605289, and #606708). It may present as a phenotype ranging from the hypoplasia of a single phalanx to the aplasia of one or more central digits (‘lobster hand’). Tandem genomic duplications on chromosome 10q24, known as the most common cause of SHFM, include the SHFM3-related dactylin gene and regulatory elements affecting the SHFM genes [1,2]. Although mostly autosomal dominant inheritance with incomplete penetrance has been observed, autosomal recessive and X-linked inheritance cases have also been described [1,3]. Monoallelic pathogenic variants in *TP63* [4], in *DLX5* [5], and in *DLX6* [6], and biallelic variants in *DLX5* [7] and in *WNT10B* [8] are known to be associated with SHFM in humans. *TP63*, a member of the p53 family of transcription factors, is critical for developing ectodermal structures, including the limbs, and its variants are often associated with ectrodactyly [9]. *DLX5* and *DLX6*, belonging to the Distal-Less Homeobox family, are expressed in the developing limb bud and play a role in the differentiation of limb structures. Mutations or changes in the expressions of these genes might disturb normal limb patterning and skeletal development [10]. *WNT10B* is involved in regulating osteoblastogenesis and is expressed in developing limbs [11]. *FGFR1*-associated congenital hypogonadotropic hypogonadism with SHFM (MIM #147950) is another presentation of this malformation [12], which can be explained by *FGFR1* expression in early limb bud expression and it being crucial for bone formation [13].

Split hand/foot malformation associated with the aplasia of long bones (SHFLD; MIM 119100) is a very rare subcategory of SHFM characterized by tibial defects (tibial hemimelia, aplasia, or dysplasia). Naveed et al. [14] conducted a genome-wide linkage analysis in a large kindred family from the United Arab Emirates and mapped two SHFLD susceptibility loci, one at 1q42.2-q43 (SHFLD1) and another at 6q14.1 (SHFLD2). The duplication of *BHLHA9* within the chromosome 17p13.3 locus has been proposed to be implicated in autosomal dominant SHFLD3 [15,16,17]. *BHLHA9* encodes a basic helix–loop–helix transcription factor, and the gene is exclusively expressed in the distal limb bud under the apical ectodermal ridge (AER). *BHLHA9* plays an essential role in normal limb development, as illustrated by knockdown experiments in zebrafish, which resulted in severe truncations of the pectoral fins [16].

Clinical findings are similar in SHFLD1, SHFLD 2, and SHFLD3, and classification was made based on genetic heterogeneity. Gollop–Wolfgang complex (GWC; MIM:228250) is defined as the combination of bifurcation of the femur with tibial agenesis with or without hand/foot malformation [18,19]. To date, more than 200 patients with GWC have been described from different ethnic backgrounds. Genetic heterogeneity, with both autosomal recessive and autosomal dominant inheritance, as well as phenotypic heterogeneity, such as unilateral or bilateral limb involvement, has been reported [20,21,22,23].

Here, we report a three-generation Turkish family with four family members presenting with the GWC phenotype with extreme clinical variability. The family tree suggests an autosomal dominant inheritance pattern with reduced penetrance. A whole-genome sequencing analysis identified a *WNT11* variant as a probable cause of the observed phenotype. In silico modelling and a luciferase assay support this disturbed Wnt11 functionality due to the detected *WNT11* c.1015G>A missense variant.

## 2. Materials and Methods

### 2.1. Patients and DNA Isolation

**Figure 1:**
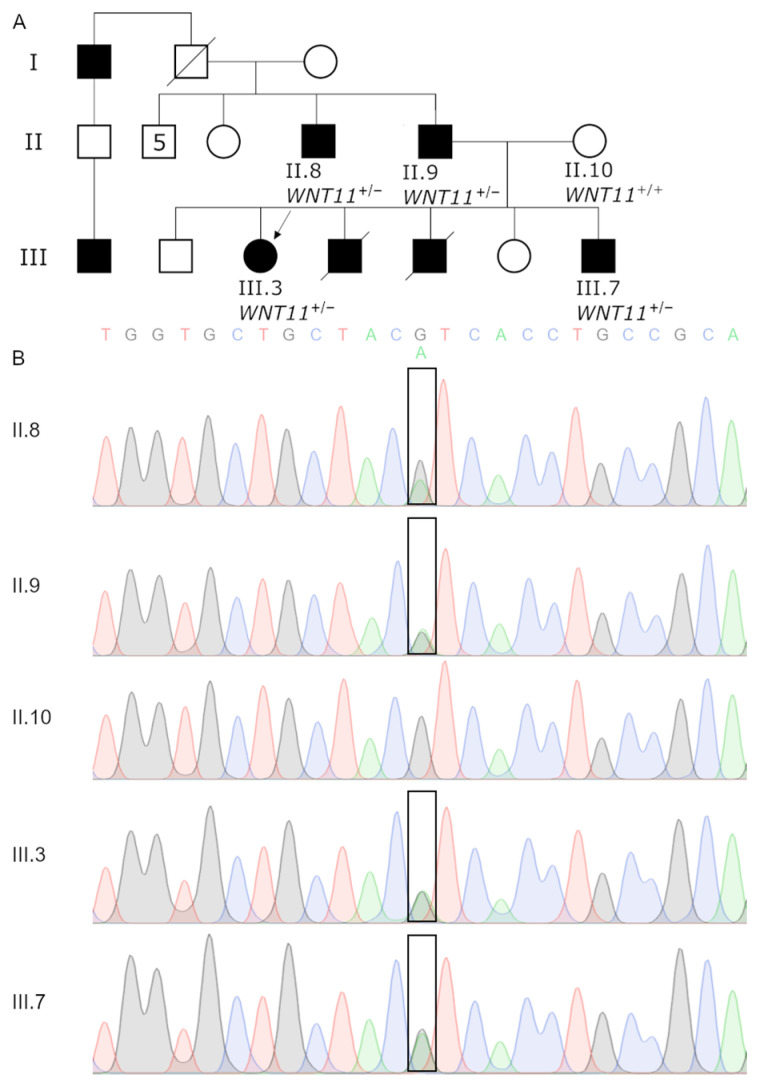
Pedigree of the family. Arrow points to the proband. Indicated samples were sequenced using whole-genome sequencing method. *WNT11*^+/+^ denotes wildtype, while *WNT11*^+/−^ heterozygous missense variant *WNT11*:c.1015G>A (**A**). The electropherograms represent Sanger sequencing of missense variant (**B**).

The four affected and one non-affected individuals from a Turkish pedigree enrolled in this study are indicated in Figure 1 (Figure 1A). All patients provided informed consent. Genomic DNA was extracted from the blood using standard techniques.

### 2.2. Whole-Genome Sequencing

Genomic DNA was sheared prior to adapter ligation. Libraries were prepared following the Kapa HTP Library Preparation Kit protocol. DNA fragments were paired-end (PE150) sequenced on the Illumina NovaSeq 6000 sequencer (Illumina, Los Angeles, CA, USA) to reach an average coverage of 30×.

### 2.3. Alignment and Variant Calling

The whole-genome data alignment and variant calling were performed by following the guidelines of Broad Institute’s best practice [24]. After quality control, raw reads were aligned to the hg38 human reference genome using BWA mem [25]. Next, quality scores were recalibrated using Base Quality Score Recalibration. Duplicates were marked using Picard, and variants were called using the Haplotypecaller from GATK [26]. After the joint-genotyping of all five family members, the obtained variants were annotated using the Variant Quality Score Recalibration. The results in the VCF format were used for subsequent analyses.

### 2.4. Identical by Descent Analysis

Identity by descent (IBD) was conducted by applying an algorithm described by Pagnamenta et al. [27], without the need to lift-over the genome version or downsampling SNP counts. A multi-sample VCF file with all sequenced family members was filtered by applying the following set of filters: a reads depth of >14, genotype quality of >30, no mendelian error, and an allelic ratio difference of >0. Next, a pair-wise analysis between all affected patients was performed. For each Single-Nucleotide Variant (SNV) in each pair, the absolute difference between the ratios of alternative variant read count and read depth was taken:

$$Ratio\;diff_{A}\;=\;\left|\frac{AD_{A}^{1}}{DP_{A}^{1}}-\frac{AD_{A}^{2}}{DP_{A}^{2}}\right|$$

where *A* denotes the current site, 1 or 2—patient from pair, *AD*—alternative variant read count (allelic depth), and *DP*—read depth.

Finally, pairs with identical genotypes were filtered out as a non-informative.

### 2.5. Variant Annotation and Prioritization

To detect the relevant variants linked to the GWC phenotype, Exomiser v13.0.1 was used with data version 2109, ReMM v0.3.1, and CADD v.1.6 [28] (the analysis parameters are available in Supplementary Table S1). Exomiser serves the dual purpose of annotating and sorting detected short variants with their level of impact on the phenotype in question and the assumed inheritance mode. The obtained sorted list of genes was shortened by discarding records that had an Exomiser Score of less than 0.5.

To asses ACMG classifications, two tools were used: InterVar [29] and Franklin (https://franklin.genoox.com, accessed on 19 January 2024). These tools provide a systematic approach to categorizing the genetic variants based on the American College of Medical Genetics and Genomics guidelines.

### 2.6. Variant Confirmation Using Sanger Sequencing

All 5 DNA samples isolated from our recruited family members’ blood were amplified via a PCR reaction with Q5 proofreading Polymerase (NEB). The primers used in this study are indicated in Supplementary Table S2. Next, the sequencing reactions with purification were carried out by an outside contractor (Eurofins, Ebersberg, Germany) to confirm the variant. The DNA sequences were analyzed using the BLAST algorithm (NCBI BLAST; http://blast.ncbi.nlm.nih.gov/Blast.cgi, accessed on 19 January 2024) querying NCBI (http://www.ncbi.nlm.nih.gov/, accessed on 19 January 2024).

### 2.7. Structural Variants

A custom pipeline was created to detect and process Structural Variants (SVs). Three tools, namely, Manta [30], Lumpy [31], and CNVnator [32], were used to call the SVs. Next, genotypes were assessed and hard filtering was applied (according to the guidelines provided by each tools’ authors). The SVs detected by multiple tools were merged. Due to variable breakends detection by each of the tools, a merging rule based on: (1) % of overlap of two regions, and (2) the bp distance between matched break ends of intervals in question had to be applied. We chose 50% reciprocal overlap and 1000 bp distance, which provided the most consistent results. Finally, each variant was annotated using a custom script to find overlapping genes, regulatory elements [33], gnomAD frequency [34], pBRIT [35], Database of Genomic Variant (DGV) [36], and IBD regions computed as described above.

### 2.8. Modelling Wnt11 Mut-Frizzled 8 Interaction

All modelling was performed using MOE (Chemical Computer Group, Canada, Montreal) [37] utilizing the amber10:EHT force field [38]. As a template, the crystal structure of Wnt3 in complex with frizzled 8 (PDB 6AHY) [39] was used. To study the conservation of the Wnt C-terminal region, a multiple-sequence alignment of human Wnt proteins was created and visualized with the weblogo tool [40].

### 2.9. Cell Transfection and Luciferase Assay

TOPFlash is a luciferase reporter containing a minimal fos promoter coupled to four Tcf-binding sites upstream of a firefly luciferase gene. FOPFlash is similar, except that its Tcf-binding sites are mutated and non-functional, serving as a negative control. Therefore, the ratio of expression from TOPFlash to expression from FOPFlash (T/F) provides a readout of canonical Wnt-specific transcriptional activity [41].

HEK 293 cells were co-transfected using TurboFect Reagent (ThermoFisher Scientific, Waltham, WA, USA) with 1 μg of DNA consisting of: (i) wt or mutant Wnt11 expression constructs (400 ng); and (ii) TOP or FOP luciferase reporter plasmid (200 ng). In order to maintain the same DNA concentration during transfection, pUC18 plasmid was used to fill up to 1 μg of the total DNA content. The transfections were carried out with Lipofectamine 2000 in 24-well plates with cells at 75% confluency. Following 24 h of incubation, the cells were washed once with PBS and used in reporter assays. These plasmids were each co-transfected with the Renilla luciferase plasmid pRLTK, which controls for transfection efficiency. The dual-luciferase system of Promega was used with a luminometer to measure the expression levels in light units. The relative luciferase activity was normalized to the β-galactosidase activity. All measurements were performed using a Tecan Infinite M200Pro reader. The statistical analysis included a non-parametric Mann–Whitney U (Wilcoxon rank-sum) test. Statistical analysis and visualization were performed using R 4.3.1 with the tidyverse 2.0.0, ggpubr 0.6.0, and rstatix 0.7.2 packages.

## 3. Results

### 3.1. Clinical Examination of GWC Patients

**Figure 2:**
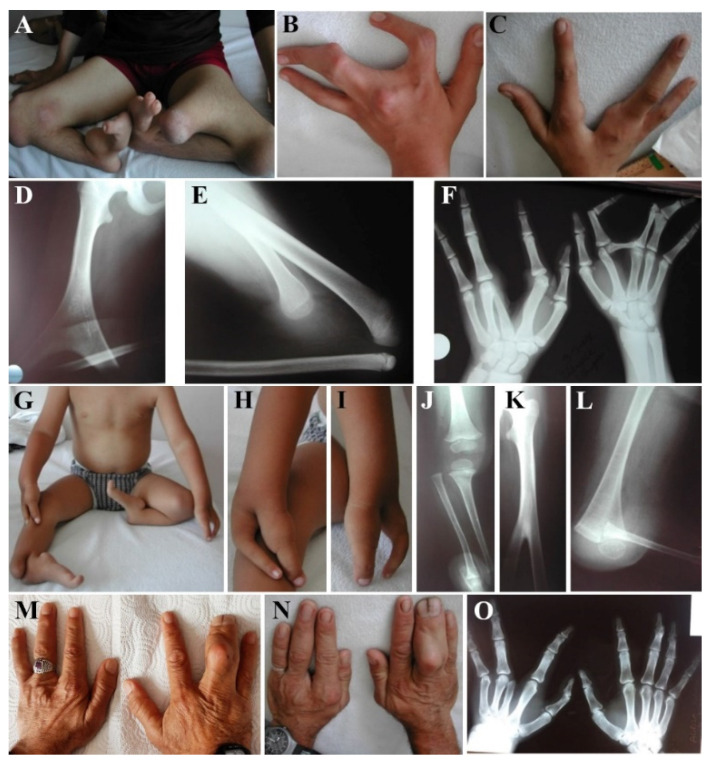
Photographs of the proband (III.3) at age of 13 years: note bilateral femur bifurcation and the fixed clubfoot deformity with only two toes on the right foot and one toe on the left foot (**A**), and bilateral split hands with the absence of the middle finger (**B**,**C**). Radiographs show bilateral bifurcation of the distal portion of the femur and absence of the tibia (**D**,**E**). Note five metacarpals bones with complete absence of the phalanges of the third finger on the left hand, and absence of the distal phalanx of the third finger, together with the accessory bone between the second and third metacarpals on the right hand (**F**). III.7 at the age of 4 years: Femoral bifurcation on the left side with bilateral only one toe on both sides (**G**) and bilateral split hands with only two digits (**H,I**). Normal femur and tibia on the right side (**J**), while femoral bifurcation and absent tibia on the left side are seen on the radiographs (**K**–**L**). Hand photographs and radiographs of II.9 (**M**) and II.8 (**N**,**O**): mild split hands with five metacarpal and four phalanges bones together four digits on the left, camptodactyly and syndactyly on the right third and fourth digits.

A 13-year-old girl (III.3) and her 4-year-old brother (III.7) were consulted for similar hand and foot deformities. Their parents were not consanguineous (Figure 1A). One of their siblings with similar features had died of bronchiolitis at 10 months of age, and the other was stillborn. III.3 and III.7 had split hands and oligosyndactyly or monodactyly of feet. While III.3 had bilateral femoral bifurcation and tibial agenesis, the same findings were present only on the left side in III.7 (Figure 2A–C,G–I). Their detailed clinical features are summarized in Table 1. Radiographs of III.3 showed bilateral bifurcation of the distal part of the femur and the absence of the tibia (Figure 2D,E). Five metacarpals on the left hand, the complete absence of the phalanges of the third finger, and the absence of the distal phalanx of the third finger were observed (Figure 2F). III.7 had unilateral (left side) femoral bifurcation and an absent tibia (Figure 2J–L). The other systemic findings of these siblings were unremarkable, and routine biochemical tests, eye examinations, hearing tests, echocardiography, and abdominal ultrasonography were also considered to be normal. The father (II.9) and paternal uncle (II.8) only had hand involvement with a split hand with four fingers on the left side and camptodactyly and syndactyly on the right side (Figure 2M–O).

### 3.2. IBD Analysis Provided Regions Shared among Affected

**Figure 3:**
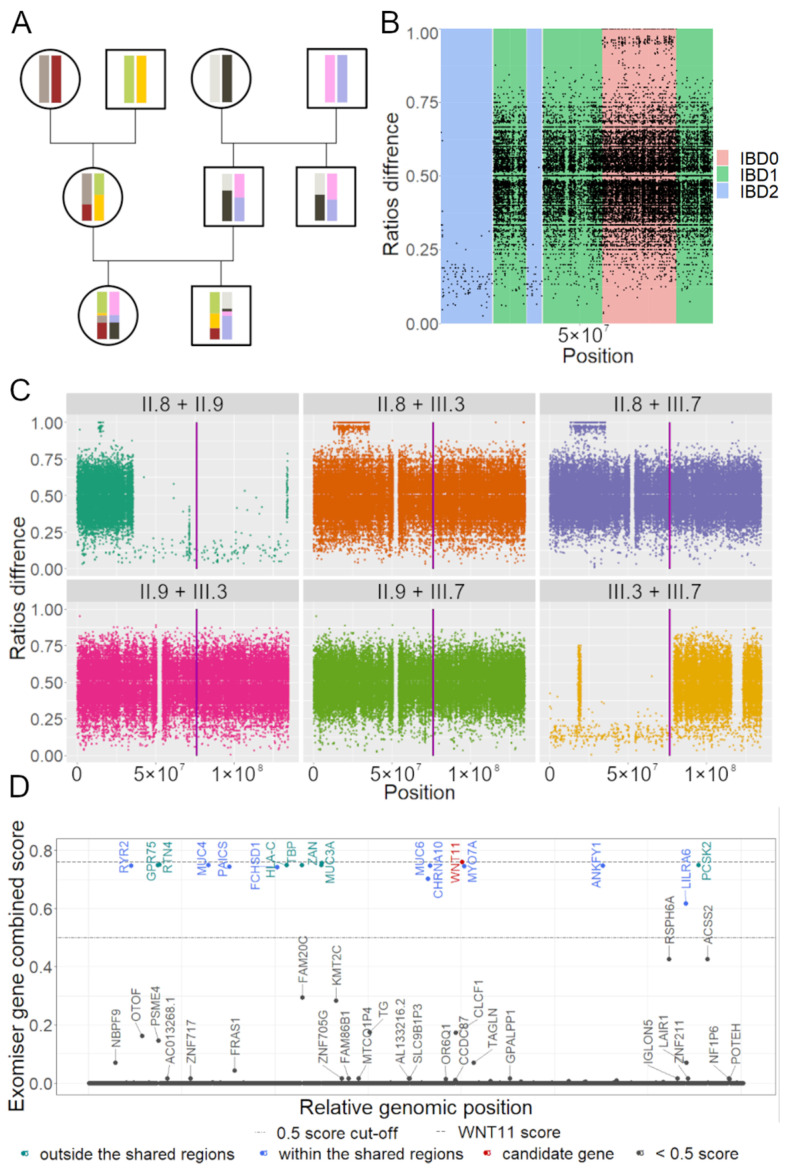
(**A**). Simplified IBD principle (based on “Pedigree, recombination and resulting IBD segments, schematic representation” image available on https://isogg.org/wiki/Identical_by_descent under CC BY-SA 3.0 license, accessed on 19 January 2024). Each individual is represented by two homologous chromosomes as bars. (**B**). IBD estimation basis. Example on chromosome 1 between III.3 and III.7. (**C**). IBD analysis was performed on chromosome 11 between all affected family members. Magenta band marks *WNT11* position. (**D**). Exomiser results. *WNT11* achieved the highest Exomiser Score. Genes with Exomiser Score of at most 0.01 are not labelled.

The occurrence of the phenotype in successive generations in both males and females is strongly suggestive of an autosomal dominant inheritance pattern. To map and identify the genetic cause, the genomes of five family members were sequenced (Figure 1a) and their genotypes were extracted. To delineate the regions in common in the affected participants but absent in the healthy relatives, we performed an identity-by descent (IBD) analysis (Figure 3A). IBD pair-wisely compares allelic ratios and hence, can reveal the regions that were inherited conjointly by the affected patients (Figure 3). Under the autosomal dominant inheritance model, we rejected regions that were not shared by at least one of the pairs between all of the affected (III.3, III.7, II.9, and II.8) with at least one allele (IBD1 or IBD2 in Figure 3B). We performed an IBD analysis on all chromosomes (Figure 3C shows results for chromosome 11 only). Across all autosomal chromosomes (hg38), we estimated that 680 regions/1229 Mb spanning 9556 protein-coding genes were shared among the affected family members.

The IBD analysis provided coarse filtering, concentrating on approximately half of the autosomal genome, thereby reducing the range of inter- and intragenic potential variants.

To identify potential candidate pathogenic variants, we resorted to similarity-to-known-phenotype-based prioritization tools. Exomiser [28] was one such tool, which identified 543 genes within the IBD regions. By applying a <0.5 Exomiser Score cut-off, the number of genes dropped to 12 across 15 SNVs/Indels (Figure 3D, Supplementary Table S3, top 500 genes detected by Exomiser in any inheritance model are provided in Supplementary Table S6). In a parallel analysis, the pBRIT prioritization tool [35] was used to evaluate the structural variants by examining the genes or their known regulators overlapping with the SVs. SVs that did not overlap with at least one phenotype-related gene or did not match the dominant inheritance mode were rejected. This approach allowed us to identify nine variants (Supplementary Table S4).

We further filtered those 12 genes and 9 SVs to put forward a single candidate gene that could explain the Gollop–Wolfgang Syndrome phenotype. Filtering included assessing variants for population frequencies, reviewing the relevant literature through Pubmed, and cross-referencing with the OMIM and Gene Ontology databases. The selected variant underwent conformation through Sanger sequencing for all four affected and one unaffected family members (Figure 1B).

The *WNT11* missense variant (access #: rs759762868; *NM_004626.2*:c.1015G>A; *NP_004617.2*:p.Val339Ile) emerged as the most suitable candidate, present in the tested affected individuals but absent in the unaffected mother (II.10). The frequency of this variant in the gnomAD v4.0 population database is extremely low (allelic frequency~0.000005582, eight counts in European, non-Finnish, and one count in South Asian). Not only is the locus predicted to be highly conserved (GERP = 4.63), but it also has high scores in most of the metrics that predict a variant functional effect on the protein (Supplementary Table S5). Furthermore, *WNT11* is known to regulate axis elongation in lower vertebrates [42]. In terms of ACMG classification, this variant aligns with several relevant criteria. Specifically, PM1 (located in a key domain involved in binding frizzled proteins), PM2 (an extremely low frequency in gnomAD), and PP3 (multiple computational tools support the variant’s deleterious effect on the gene). Overall, these classifications lead to a conclusion that the variant is of uncertain significance (ACMG class 3).

### 3.3. Wnt11 Variant Influences Interaction with Frizzled 8 Protein

**Figure 4:**
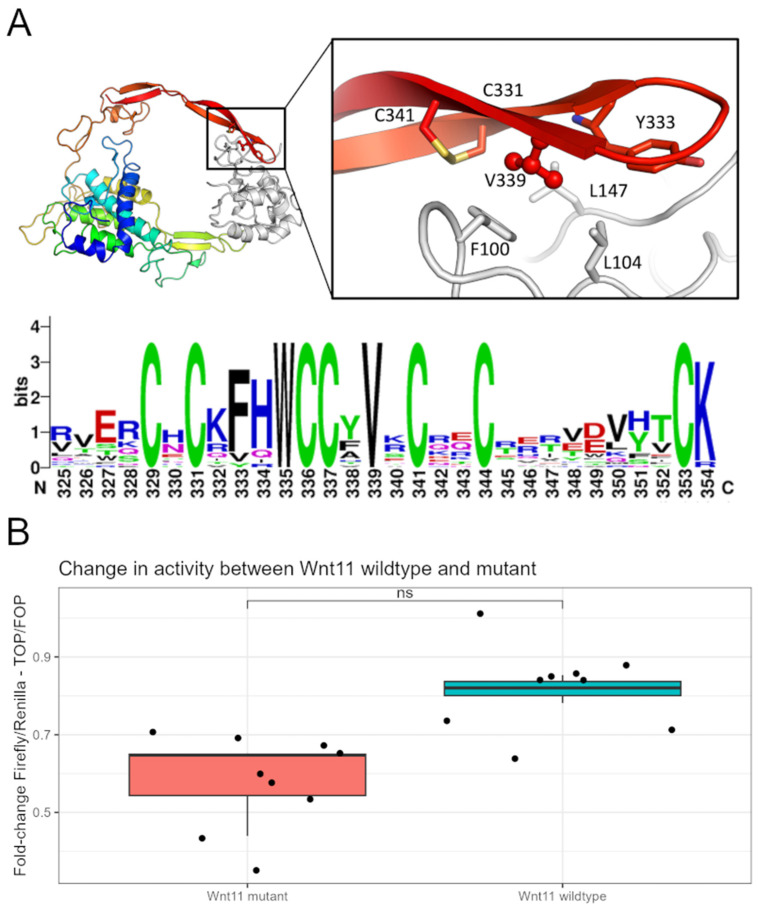
(**A**). The Wnt11 is shown as a cartoon colored blue to red from N to C terminus. The Frizzled8 protein is depicted in white. V339 (ball and stick) is buried in a hydrophobic patch. The Sequence logo (created using Weblog) of the C-terminal sequence of the human Wnt family reveals perfect conservation of the V339 residue (numbering according to Wnt11). (**B**). The results of transactivation of Wnt reporter plasmid by Wnt11 wt or mt on the TOP/FOP activity. Results of luciferase assay are presented as Relative Light Units (RLUs) of tested variants normalized to renilla activity. Graph shows results of representative experiment. Data represent the boxplot of triplicate samples. *p*-value = 0.05 with effect size (r) of 0.8.

To investigate the influence of the LV variant on a structural level, a homology model of Wnt11 bound to a frizzled protein was created. The human Wnt11 sequence was obtained from Uniprot (O96014) and the homology modelling module of MOE was modelled with PDB entry 6AHY as a template structure with its energy minimized. The Frizzled 8 protein was used as an environment to create a complex structure. This homology model reveals a tight interaction between V339 and residues F100, L104, and L147 on the frizzled 8 protein, as well as Wnt11 flanking residues (Figure 4A). C331, Y333, and C341 Replacement of the valine with a larger isoleucine would, therefore, sterically prevent the correct binding to the frizzled proteins. The essential role for the interaction of V in the Wnt family of proteins is further illustrated by the perfect conservation of the residue within the human Wnt family.

### 3.4. Wnt11 Variant Weakens a Cellular Response to the Ligand

The Wnt signaling pathways are integral to numerous cellular processes, and disruptions in ligand–receptor interactions can lead to altered cellular responses [43,44]. One of the canonical Wnt receptors is the family of frizzled 7-pass transmembrane proteins. Following the receptor binding, the ensuing pathway activation is complex and multifaceted. There are several ways to classify Wnt signaling and one of them divides it into β-catenin-dependent or -independent events. Wnt11 is typically associated with planar cell polarity signaling, but is also known to regulate β-catenin-dependent signaling [45,46,47]. This pathway is typically activated when a Wnt protein binds to the frizzled receptor on the cell surface. This binding leads to the stabilization and accumulation of β-catenin in the cytoplasm, which then translocates to the nucleus. In the nucleus, β-catenin forms a complex with TCF/LEF transcription factors, leading to the transcription of target genes. The missense variant 1015G>A in *WNT11* is hypothesized to weaken its interaction with the cognate receptor, leading to decreases in cellular response. Thus, we employed a classical Wnt reporter assay based on the TOP/FOP system to elucidate its potential effect [48]. The reporter plasmid was co-transfected into HEK293 cells with the Wnt11 wt or variant expression plasmid. Following cell lysis, we measured the luciferase activity of the TOP reporter and normalized it to the renilla activity. We could show that the Wnt11 variant decreased the reporter response. The abovementioned outcome was confirmed by three independent experiments, performed in triplicate at 1–2-week intervals (Figure 4B). The wild type displayed modestly higher activity compared to the variant version, with this difference reaching a *p*-value of 0.05 in the Mann–Whitney test. While this value suggests a borderline statistical significance, it indicates that the Wnt11 variant may lead to a mild attenuation in signaling capacity, as evidenced by the reduced transactivation activity in the assay.

## 4. Discussion

We described a large family with two siblings with split hands and mono/oligodactyly of the feet, accompanied by distal femoral bifurcation and ipsilateral absence of the tibia, which is clinically consistent with Gollop–Wolfgang complex. Both their father (II.9) and paternal uncle (II.8) also presented with split of the hands. This large pedigree corresponds to the incomplete penetrance of an autosomal dominant GWC (Figure 1). We identified a *WNT11* variant as the most likely cause of the patients’ phenotype.

To detect possible variants, we constructed a pipeline consisting of three principal components: (1) calling SNVs/indels variants using standard Broad Institute’s Best Practices followed by structural variant detection, (2) Identity by Descent analysis, and (3) the annotation and prioritization of the called variants. The analysis identified 24 plausible variants, however, after a further review of the literature, we selected only 1 strong candidate located in *WNT11*. The low prevalence in the population, along with the possible incomplete penetrance in this family, suggests that *WNT11*:c.1015G>A can be associated with the molecular etiology of GWC. This variant leads to the substitution of 339 valine to isoleucine. The position of 339 valine is present in multiple Wnt ligands, suggesting its potentially important role in interaction with the Wnt receptor. We demonstrate *WNT11* variants to have a lower transactivation of the Wnt reporter than the wild type, supporting the notion that the substitution of 339 valine sterically interferes with the correct binding of *WNT11* to the frizzled receptor. *WNT11* itself is a component of canonical and non-canonical Wnt pathways that orchestrate osteoblast differentiation and mineralization [49] across multiple species. *WNT11* is expressed in the mesenchyme and apical ectodermal ridge during murine limb bud development (E9.5 to E11.5) [50,51], as well as in chick embryos with specific spatiotemporal patterns in the forming limb bud [52]. In zebrafish, wnt11f2 plays a vital role in cartilage development, and mutations in this gene lead to craniofacial malformations [53], with variable severity of the defects [53]. Due to a high conservation of Wnt11, zebrafish’s *wnt11f2* has been suggested as a suitable model [49]. Heisenberg et al. recognized two specific mutations, tx226 (c.669G>A p.Trp 223) and tz216 (c.463G>T p.Gly 155), while Sisson et al. [53] showed that 4 dpf fish carrying a tz216 mutation had deformed cartilage elements, especially the ceratohyal and Meckel’s cartilage [49].

Several other mutations of *WNT11* have been described. Caetano da Silva et al. found three unrelated patients with monogenic early-onset osteoporosis (EOOP) associated with *WNT11′*s loss-of-function. Patient 1 had a low bone mineral density with the *NM_004626.2*:c.677_678dup p.Leu227Glyfs*22 variant, while patient 2 and patient 3 had two heterozygous *WNT11* missense variants: *NM_004626.2*:c.217G > A p.Ala73Thr and *NM_004626.2*:c.865G > A p.Val289Met, respectively, both with bone fragility [54]. Only patient 2 had a confirmed inheritance of the heterozygous mutation from her mother, who herself experienced multiple osteoporotic fractures, indicating a potential familial predisposition. Unfortunately, the studied cases of EOOP, including this familial instance, have not provided evidence of incomplete penetrance. The results of the in silico modeling and in vitro assay suggest a modest negative change in Wnt11 signaling potential. This is in line with the observations described above. Additionally, it is important to remember that Wnt11 is expressed throughout development, starting with gastrulation. Therefore, even mild differences in protein activity may lead to profound, phenotypic changes.

GWC and EOOP do not share phenotypical similarities. EOOP primarily affects bone density and fragility, whereas GWC is characterized by more complex limb malformations. The Wnt pathway is involved in both the early development of limbs [55] and the maintenance of bone density [54]. Thus, pathological variants in *WNT11* might disrupt the signaling necessary for proper limb formation, leading to the characteristic malformations in GWC. On the other hand, in EOOP, the same pathway’s disruption affects bone remodeling and density, as Wnt signaling is crucial in osteoblast function and bone mineralization. The variability in the specific pathological variants within the *WNT11* gene can lead to different effects on the protein’s function and its interaction with the Wnt signaling pathway, contributing to the manifestation of these distinct disorders.

It has been suggested that there is also autosomal recessive and dominant inheritance in GWC. In studies presenting GWC cases with autosomal recessive inheritance, it has been reported that the patients did not have a split of the hands, but also had severe cardiac and renal involvement [55]. A genetic evaluation of large families with GWC revealed an autosomal dominant inheritance with reduced penetrance and variable expression [3]. In the family we studied, the manifestation of GWC showcases a clear pattern of incomplete penetrance and variable expressivity. III.3 and III.7 both presented with split hands and femoral bifurcation with tibial agenesis. However, the severity of their conditions varied: while III.3 exhibited a bilateral split hand with four digits, femoral bifurcation, and tibial agenesis, III.7 had a bilateral split hand with two digits and unilaterally femoral bifurcation with tibial agenesis. II.9 and II.8, also only showing signs of the complex with split hands and syndactyly, displayed much milder symptoms compared to the children.

## 5. Conclusions

In conclusion, we performed a set of computational and in vitro functional analyses to discover the molecular cause of Gollop–Wolfgang Syndrome. The missense variant c.1015G>A in the *WNT11* gene was detected as most likely being responsible for the observed GWC phenotype in one family. However, further studies, preferentially using an animal model(s), would provide more insight into the role of *WNT11* in GWC. Additionally, to more comprehensively understand the genetic landscape of GWC and validate the involvement of *WNT11*, future research involving a larger cohort of GWC patients is needed.

## Figures and Tables

**Table 1:** Skeletal features of the patients.

Patient Numbers	III.3	III.7	II.9	II.8
Split of Hand	Bilateral	Bilateral	Unilateral (Left)	Unilateral (Left)
Number of fingers				
Right	Four	Two	Five	Five
Left	Four	Two	Four	Four
Syndactyly of finger	Right (between second and third finger)	-	Right (between second and third finger)	Right (between second and third finger)
Femur bifurcation	Bilateral	Unilateral (left)	-	-
Absent tibia	Bilateral	Unilateral (left)	-	-
Number of toes				
Right	Two	One	Five Five	Five Five
Left	One	One		
Syndactyly of foot	Right (between first and second finger)	-	-	-

## Data Availability

Data are not publicly available and will be available upon reasonable request from the corresponding author, as the clinical reports contain information that could compromise the privacy of involved individuals.

## Supplementary Materials

The following supporting information can be downloaded at: https://www.mdpi.com/article/10.3390/genes15010129/s1, Supplementary Figure S1: Structural variants detection pipeline; Supplementary Figure S2: Visual representation of (A) merging two genomic intervals by % overlap and bp distance between two furthest break ends. (B) providing consensus region, based on 50% overlap and 1000 bp; Supplementary Figure S3: Complete overview on combined IBD; Supplementary Table 1: Exomiser settings; Supplementary Table S2: Primers used for sanger sequencing conformation; Supplementary Table S3: Filtered SNVs and small indels detected by the pipeline; Supplementary Table S4: Filtered Structural variants detected by the pipeline; Supplementary Table S5: Selected metrics of *WNT11* mutation; Supplementary Table S6: Exomiser top 500 genes variants.
