# Supplementary material for: Gollop–Wolfgang Complex Is Associated with a Monoallelic Variation in WNT11
Source: Genes (Basel). 2024 Jan 20;15(1):129. doi: 10.3390/genes15010129 (PMC10815061; doi:10.3390/genes15010129)
Supplement: Supplementary file 1 [file genes-15-00129-s001.zip › Supplementary figures.pdf]

Supplementary figures

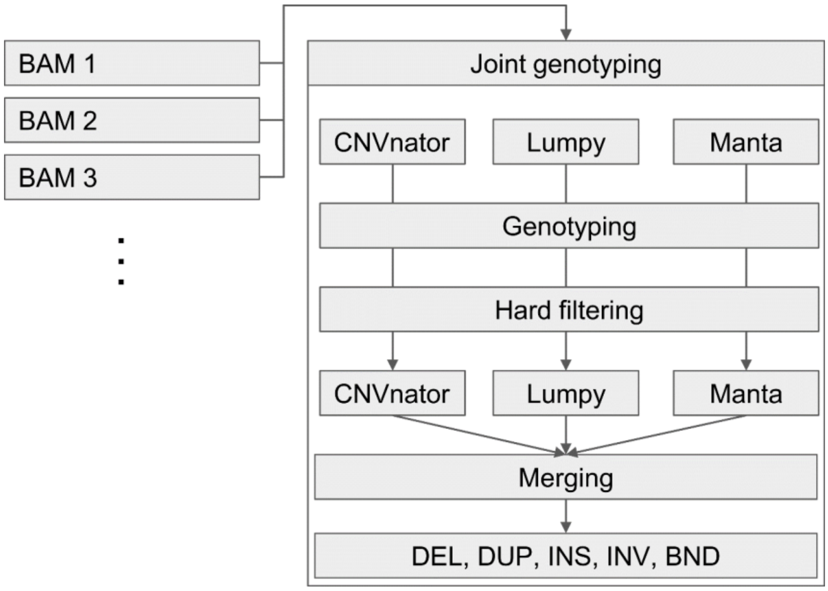

Supplementary figure 1. Structural variants detection pipeline

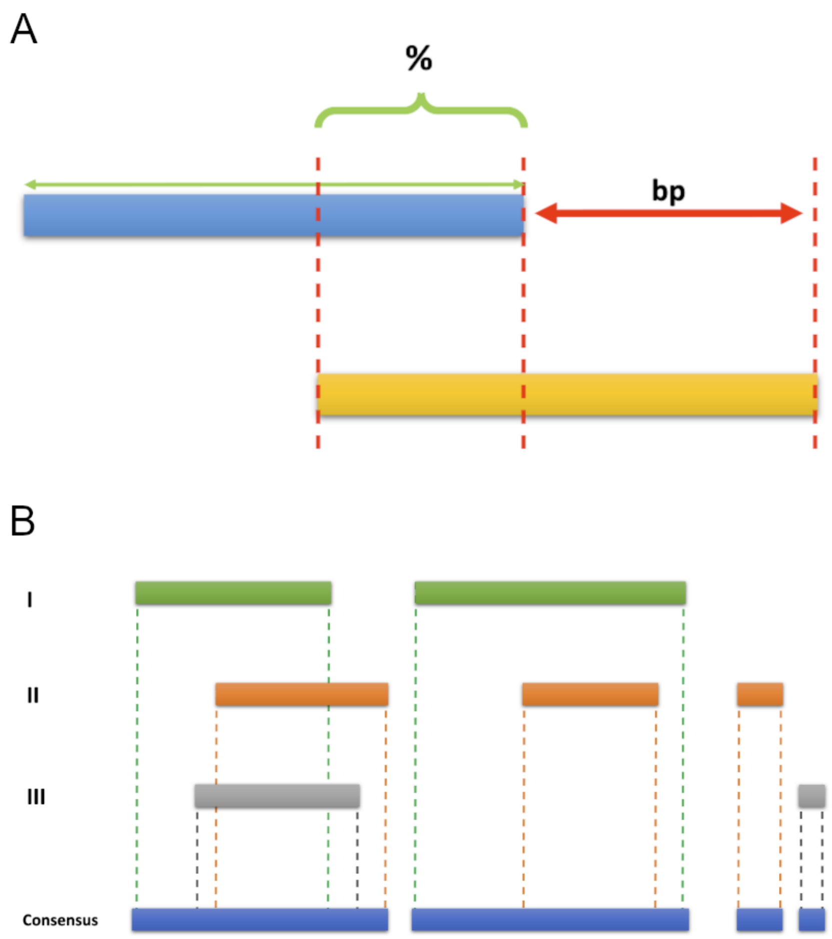

Supplementary figure 2. Visual representation of (A) merging two genomic intervals by % overlap and bp distance between two furthest break ends. (B) providing consensus region, based on 50% overlap and 1000 bp.

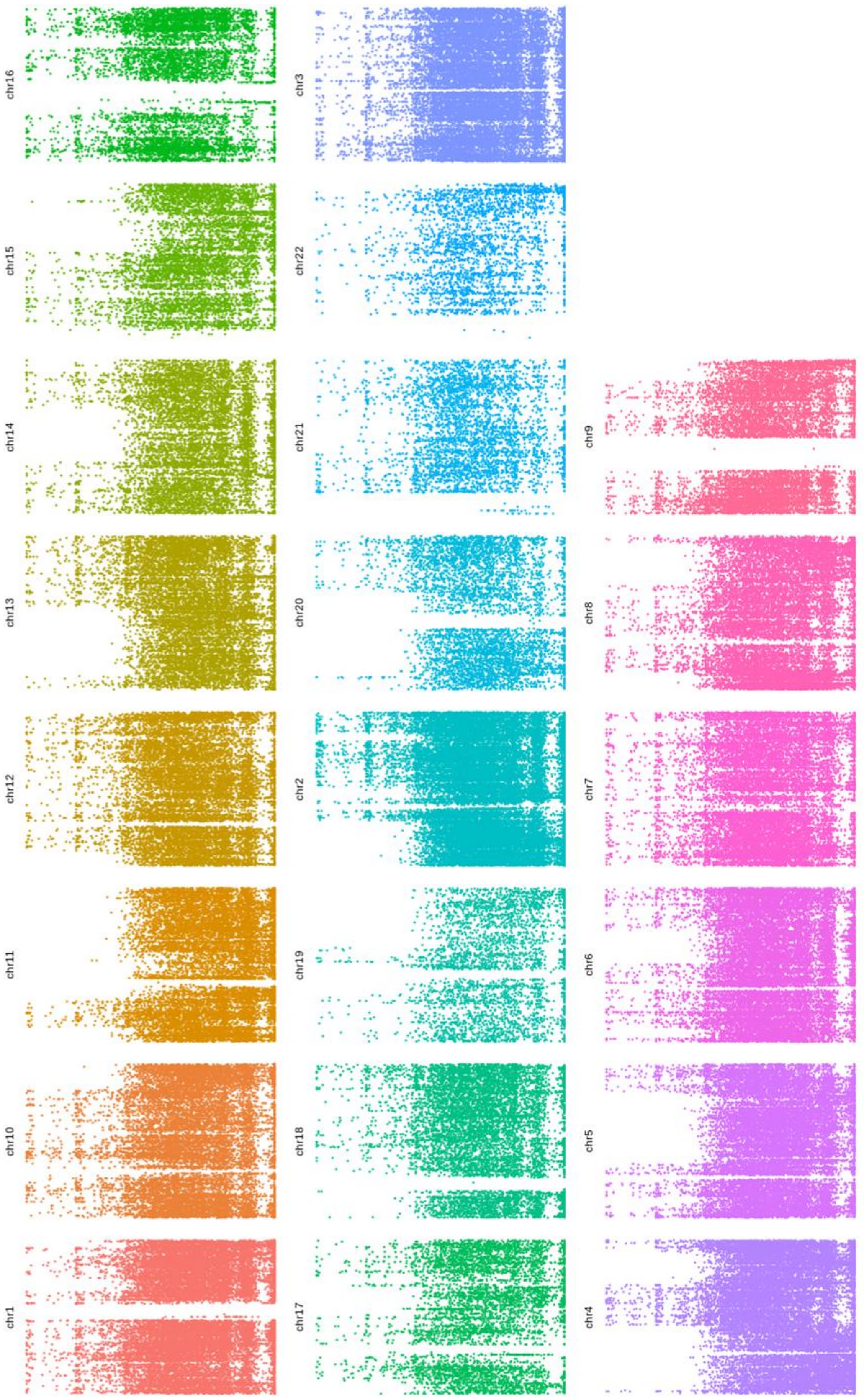

Supplementary figure 3. Complete overview on combined IBD.
